# Association of triglyceride-glucose-body mass index with all-cause mortality among individuals with cardiovascular disease: results from NHANES

**DOI:** 10.3389/fendo.2025.1529004

**Published:** 2025-01-27

**Authors:** Yiaoran Sun, Yuecheng Hu

**Affiliations:** ^1^ School of Basic Medicine, Tianjin Medical University, Tianjin, China; ^2^ Clinical School of Thoracic, Tianjin Medical University, Tianjin, China; ^3^ Department of Cardiology, Tianjin Chest Hospital, Tianjin, China

**Keywords:** NHANES, TyG-BMI, cardiovascular disease, TyG index, CVD (cardio vascular disease)

## Abstract

**Background:**

The objective of this study was to explore the relationship between the triglyceride-glucose-body mass index (TyG-BMI) and all-cause mortality rate and to determine valuable predictive factors for the survival status of patients with cardiovascular disease (CVD).

**Methods:**

Conduct a study on CVD patients in the NHANES database from 2007 to 2016. Patients were divided into four groups based on the weighted quartiles of TyG-BMI. Kaplan-Meier curves, Cox regression, and restricted cubic spline (RCS) were used to analyze the correlation between this index and all-cause mortality. Receiver operating characteristic (ROC) curves were used to evaluate its predictive ability, sensitivity, and specificity.

**Results:**

This study included 1085 patients, and revealed significant differences in survival rates among patients with different TyG-BMI levels. Patients in the higher TyG-BMI group have a lower mortality risk, yet there is no evident non-linear relationship. The ROC curve indicates that this indicator can serve as a predictive value for mortality in CVD patients, demonstrating good sensitivity and specificity.

**Conclusion:**

This study found a significant association between TyG-BMI index and all-cause mortality in patients with CVD. TyG-BMI can be used as a predictive indicator of all-cause mortality in CVD patients.

## Introduction

Cardiovascular disease(CVD), as a serious health problem, is the cause of death for one-third of the global population (1). Coronary heart disease(CHD) and heart failure(HF), as the most common cardiovascular diseases in clinical practice, can cause malignant arrhythmias, cardiogenic shock, and even sudden death. Its high incidence rate, disability and mortality rate pose a serious threat to the global health system (2, 3). Although the treatment of cardiovascular disease has made good progress, the mortality rate of patients has not been reduced to a satisfactory level. Therefore, early identification and intervention of risk factors that affect mortality are very important for the world’s public health system.

Insulin resistance (IR) is a condition in which the body’s sensitivity to insulin decreases, leading to ineffective uptake and utilization of blood glucose by target tissues and cells (4). In recent years, many studies have shown that IR is one of the important risk factors for cardiovascular, diabetes, kidney disease and liver disease (5–7). Triglyceride-glucose (TyG) and triglyceride glucose-body mass index (TyG-BMI), due to their ease of measurement and non-invasive nature, are replacing the high insulin normal glucose clamp test and Homeostasis Model Assessment of Insulin Resistance (HOMA-IR) as simple indicators for IR testing (8–10).

Studies have shown that there may be varying degrees of correlation between the TyG index and mortality rates among critically ill patients with acute myocardial infarction at different stages (11, 12). However, few studies have evaluated the relationship between the TyG-BMI index and all-cause mortality in patients with general cardiovascular diseases. Currently, there is still a lack of evidence to support the use of the TyG-BMI index as a long-term predictor of all-cause mortality risk in these patients. Therefore, this study aims to investigate the correlation and predictive value of the TyG-BMI index with all-cause mortality in CVD patients.

## Methods

### Study population

**Figure 1:**
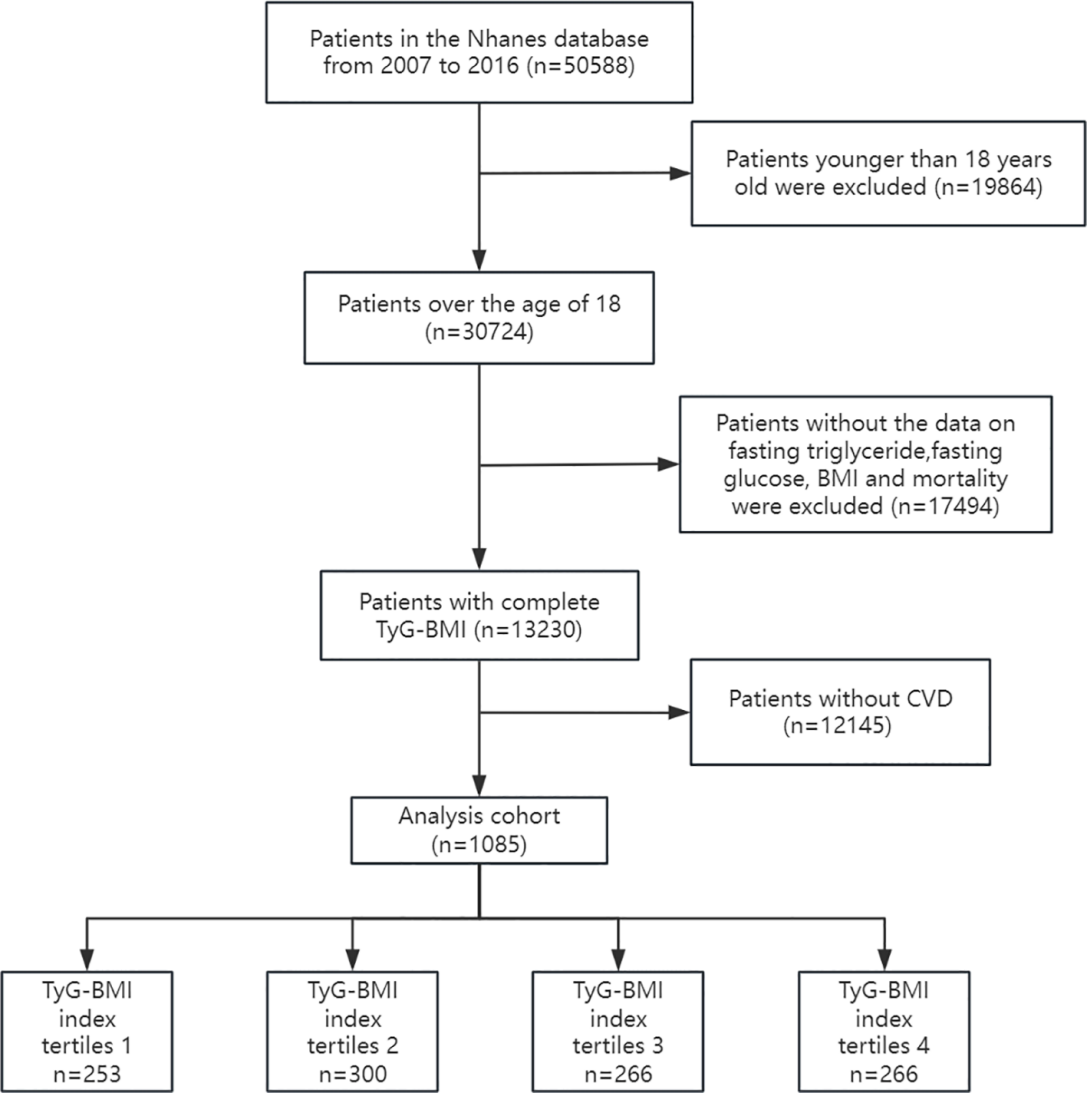
Flow chart.

Data were obtained from the National Health and Nutrition Examination Survey (NHANES 2007–2016) and the National Death Index (NDI). This database collects the health and nutrition status, examination results, and follow-up outcomes of different populations in the United State. The NHANES protocol has obtained informed written consent from all participants in the study. Our study on the correlation between TyG-BMI index and all-cause mortality in cardiovascular disease patients used NHANES data from 2007 to 2016. The following patient groups were excluded from the investigation: (1) Participants under the age of 18; (2) Participants lacking fasting blood glucose, triglycerides, BMI, and mortality outcome data; (3) Participants who do not suffer from CVD (coronary heart disease, angina pectoris, myocardial infarction, and heart failure). The study ultimately included 1085 participants. The flowchart for research is shown in **Figure 1**:

### Data collection

The TyG-BMI index was designated as the primary variable of this investigation. Extract baseline data of patients from the database, Including demographic data [age, gender, race, education level, smoking], vital signs [BMI, systolic blood pressure SBP, diastolic blood pressure DBP], laboratory parameters [triglyceride, cholesterol, urinary creatinine, blood sugar, insulin], complications [diabetes, kidney disease]. To reduce potential bias, this study excluded variables with missing values exceeding 15%. Use the “mice” package in R software to handle missing data.

### Definition and clinical outcomes

The TyG-BMI index was calculated using the following equation: In [TG (mg/dL) × FBG (mg/dL)/2] × BMI (11). CVD patients are those who have been informed by their doctors that they have coronary heart disease/angina pectoris/myocardial infarction/heart failure according to NHANES survey data. The primary endpoint of this study is all-cause mortality, The follow-up period starts from the date of the interview and ends on December 31, 2019.

### Statistical analysis

Stratify population characteristics based on the weighted quartiles of the TyG-BMI index, with categorical variables being weighted percentages and continuous variables being weighted means and standard deviations. Compare the four groups using analysis of variance or Kruskal-Wallis test for continuous variables, and the chi-square test for categorical variables. To investigate the effect of different TyG-BMI index levels on all-cause mortality, Kaplan-Meier survival analysis was conducted and log-rank test was used for inter group comparison.

Evaluating the relationship between different TyG-BMI index and survival status using a combination of univariate and multivariate Cox proportional hazards models. We adjusted for different covariates and constructed three regression models. Model 1 is an unadjusted reference; Model 2 adjusted for age, gender, and race; Model 3, as a comprehensive adjustment, added smoking SBP, DBP, TC, insulin, covariates such as diabetes and kidney disease. Quantify the association between TyG BMI index and mortality rate by calculating the hazard ratios (HR) and 95% confidence intervals for different models. By establishing a restricted cubic spline RCS regression model, we explored the nonlinear relationships between variables (three nodes were selected in this study). Receiver operating characteristic (ROC) curves were used for diagnostic value analysis, and the area under the curve, as measured by the C-statistic, was computed to quantify the predictive power of TyG-BMI. The data analysis is conducted using R software (version 4.3.2). Use a P-value less than 0.05 to determine statistical significance.

## Results

### Population characteristics stratified by TyG BMI index

As shown in **Table 1**, according to the weighted TyG-BMI index quartile level (Q1:<219.2; Q2:219.2~263.2; Q3:263.2~312.0;Q4:>312.0), Stratify the patients. The results showed that there were certain differences in age, gender, smoking, blood pressure, and comorbidities among the groups of patients. The patients in the group with the highest TyG-BMI index are younger, less male, have higher education levels, and have significantly higher BMI and serum insulin levels than the other three groups.

**Table 1:** Characteristics of participants in the NHANES(2007-2016).

Variables	TyG-BMI index levels				P-Value
	Q1	Q2	Q3	Q4	
**n**	253	300	266	266	
**Age, years**	67.26 ± 12.93	68.88 ± 12.80	67.06 ± 12.45	62.35 ± 11.57	0.002
Gender, n(%)					0.014
Male	138 (50.4)	197 (65.9)	164 (62.7)	124 (51.2)	
Female	115 (49.6)	103 (34.1)	102 (37.3)	142 (48.8)	
Race, n(%)					0.089
Mexican American	15 (2.4)	29 (3.6)	27 (4.5)	34 (5.8)	
Non-Hispanic Black	45 (8.8)	57 (12.3)	40 (8.9)	51 (10.7)	
Non-Hispanic White	150 (75.0)	162 (73.9)	161 (78.0)	141 (75.0)	
Other Hispanic	19 (3.6)	33 (5.2)	25 (3.5)	35 (5.5)	
Other Race	24 (10.2)	19 (4.9)	13 (5.1)	5 (3.1)	
Education, n(%)					0.438
Below high school	87 (27.4)	97 (24.3)	101 (30.1)	94 (22.8)	
High School or above	166 (72.6)	203 (75.7)	165 (69.9)	172 (77.2)	
Smoke, n(%)					0.002
Yes	76 (35.0)	52 (15.9)	43 (17.2)	54 (23.5)	
No	177 (65.0)	248 (84.1)	223 (82.8)	212 (76.5)	
**BMI, kg/m2**	23.08 ± 2.32	27.63 ± 2.03	31.61 ± 2.37	40.04 ± 6.11	<0.001
**SBP, mmHg**	127.60 ± 21.68	131.02 ± 21.28	130.33 ± 19.69	128.62 ± 17.25	0.470
**DBP, mmHg**	63.31 ± 16.83	65.94 ± 16.02	67.98 ± 14.42	69.84 ± 12.65	0.005
**Ucr, mg/dl**	96.81 ± 57.98	114.94 ± 65.53	124.32 ± 77.60	123.88 ± 64.25	0.001
**Cholesterol, mg/dl**	172.44 ± 40.29	170.79 ± 39.97	178.08 ± 42.11	183.25 ± 46.95	0.073
**TG, mg/dl**	85.72 ± 37.61	117.09 ± 55.18	165.12 ± 100.10	203.38 ± 197.04	<0.001
**Fasting Glucose, mg/dl**	103.67 ± 20.97	114.00 ± 32.81	125.61 ± 34.73	141.38 ± 60.18	<0.001
**Insulin(uU/mL)**	7.71 ± 5.16	11.23 ± 6.28	17.06 ± 11.71	26.82 ± 23.25	<0.001
Diabetes, n(%)					<0.001
Yes	45 (15.6)	85 (24.6)	109 (33.9)	137 (47.7)	
No	201 (82.5)	205 (72.3)	144 (59.4)	116 (47.1)	
Borderline	7 (1.9)	10 (3.1)	13 (6.7)	13 (5.2)	
Kidney, n(%)					0.632
Yes	24 (7.4)	32 (9.5)	37 (11.3)	33 (10.2)	
No	229 (92.6)	267 (90.5)	229 (88.7)	231 (89.8)	

BMI, body mass index; SBP, systolic blood pressure; DBP, diastolic blood pressure; TG, triglyceride; Ucr, urinary creatinine.

### Kaplan-Meier analysis

**Figure 2:**
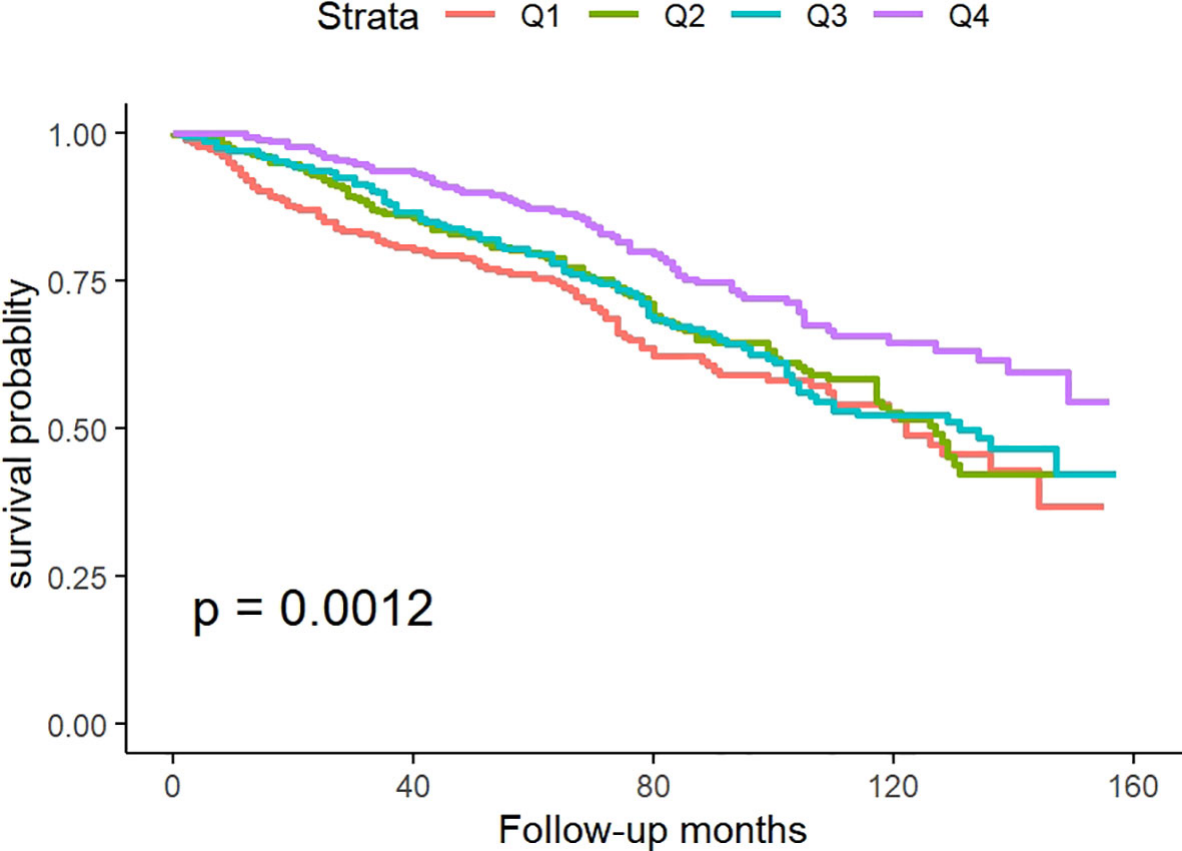
Kaplan-Meier.

**Figure 2** illustrates Kaplan-Meier survival analysis curves evaluating mortality rates between different TyG-BMI groups. The results showed that there was a significant difference in the survival rate within 160 months among the four groups of patients (p for log-rank test<0.05), and the survival rate in Q4 was significantly higher than the other three groups.

### Cox regression analysis

The correlation between TyG-BMI and all-cause mortality was analyzed using Cox proportional hazards regression, and the results are shown in **Table 2**. Using Q1 as the standard [Q1: HR=1], the Q4 group in Model 1 was significantly associated with lower all-cause mortality (HR 0.55, 95% CI 0.41-0.75). In Model 2, there were no significant differences among the groups. In Model 3, both the Q2 group (HR 0.75, 95% CI 0.57-0.98) and the Q4 group (HR 0.65, 95% CI 0.47-0.90) were associated with lower all-cause mortality rates. These results align with those obtained from the K-M survival curve.

**Table 2:** Cox.

Variables	Model 1		Model 2		Model 3	
	HR (95%CI)	P-value	HR (95%CI)	P-value	HR (95%CI)	P-value
TyG-BMI						
Q1	Ref.		Ref.		Ref.	
Q2	0.86 (0.66-1.12)	0.270	0.76 (0.58-1.00)	0.048	0.75 (0.57-0.98)	0.036
Q3	0.85 (0.64-1.12)	0.242	0.85 (0.64-1.12)	0.236	0.77 (0.58-1.02)	0.072
Q4	0.55 (0.41-0.75)	<0.001	0.76 (0.56-1.04)	0.082	0.65 (0.47-0.90)	0.010

Model 1 is unadjusted; Model 2 adjusted for age, gender, and race; Model 3 adjusted for age, gender, race, smoke, SBP, DBP, TC, insulin, diabetes and kidney disease.

### Restricted cubic splines

**Figure 3:**
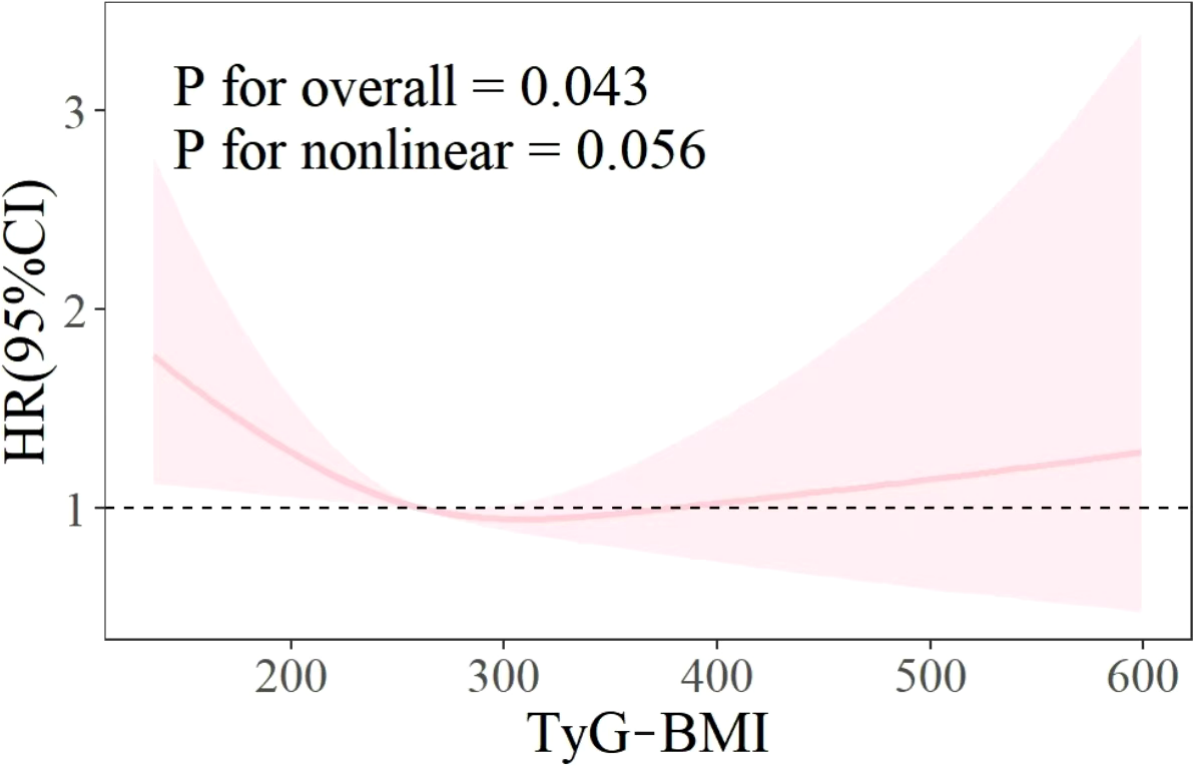
RCS.

As illustrated in **Figure 3**, the RCS curve shows the relationship between the TyG BMI index and all-cause mortality in patients with CVD. The results showed that there was no non-linear relationship between TyG BMI index and all-cause mortality rate (P for overall<0.05, P for nonlinear>0.05).

### Sensitivity and specificity analysis

**Figure 4:**
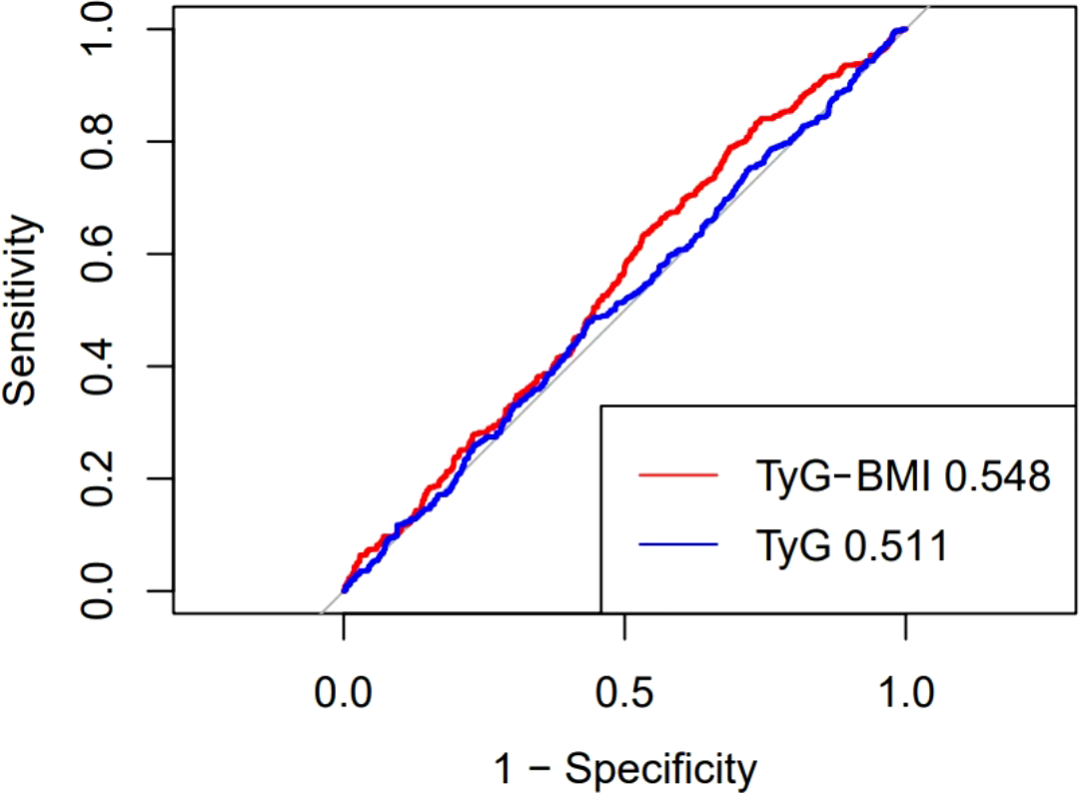
ROC.

The ROC curve is shown in **Figure 4**, which is used to evaluate the sensitivity and specificity of TyG-BMI index as a diagnostic tool for all-cause mortality. Compared with the TyG index alone, TyG-BMI has a stronger predictive ability for CVD patients. TyG-BMI (AUC 0.548, 95%CI 0.513-0.583) vs. TyG (AUC 0.511, 95%CI 0.476-0.547). This study used Youden Index to determine the optimal critical point of TyG-BMI at 304.45, with a sensitivity of 79.0% and a specificity of 68.6%.

## Discussion

The objective of this study was to explore the relationship between the TyG-BMI index and all-cause mortality rate and to determine valuable predictive factors for the survival status of patients with CVD. Using Kaplan-Meier survival analysis, univariate and multivariate Cox proportional hazards regression models, RCS curve analysis, etc., it was determined that TyG-BMI index can be used as a predictive indicator for all-cause mortality in CVD patients, and compared with TyG, it has better predictive ability, higher sensitivity, and specificity. In addition, the survival analysis results indicate that patients with TyG-BMI>312 have the lowest all-cause mortality rate in both unadjusted and fully adjusted models. This means that non critical CVD patients may be able to achieve a lower risk of death by appropriately increasing their TyG-BMI. However, although the RCS curve results do not support a nonlinear relationship between the two (p=0.056), by fitting the curve trend in the graph, it can be seen that excessively high TyG-BMI will also increase the risk of all-cause mortality. Therefore, excessively increasing or decreasing TyG-BMI is not advisable behavior. This will help develop new guidelines for reducing the risk of death.

In recent years, TyG and its related indicators have been widely studied in various systemic diseases (13–15). TyG-BMI, as an effective alternative indicator of insulin resistance, is a key factor in evaluating type 2 diabetes, obesity, metabolic abnormalities, and dyslipidemia (16–19). However, the relationship between TyG and its related index and CVD is still inconclusive, and the existing research results are roughly the following two: the first conclusion is that there is a linear and positive correlation between TyG related index and the incidence, disease progression and mortality of CVD (20–23), which means that TyG and TyG-BMI are absolute risk factors for CVD (24, 25). Another conclusion is that the TyG related index has a nonlinear or U-shaped relationship with the occurrence and progression of CVD (26–28), which means that too high or too low TyG and TyG-BMI may lead to an increased risk of death in patients with CVD. This may be influenced by various factors, including the level of insulin resistance, inflammatory response, oxidative stress, and vascular endothelial function. Consequently, an excessively low TyG may also elevate the risk of cardiovascular mortality (29, 30). The findings of this study align with this conclusion. Furthermore, the RCS curve does not indicate the dispersion of the data. This could be attributed to the limited number of CVD patients, varying disease complexities, the configuration of RCS curve parameters, and model selection.

Both conclusions acknowledge the risk posed by a high TyG-related index to CVD. Prospective studies have indicated that a high TyG-BMI ratio can exacerbate the mortality risk among CVD patients (31), potentially due to insulin resistance-induced glucose metabolism disorders, lipotoxicity, and excessive inflammatory response (32). This study will not delve further into this matter, but will solely focus on the risk associated with an excessively low TyG-BMI index.

On the one hand, lower fasting blood glucose can significantly decrease TyG, and repeated hypoglycemia can cause serious irreversible damage to multiple organs, mainly the brain, leading to an increased risk of multifactorial cardiovascular and cerebrovascular death (33, 34). On the other hand, a low BMI may be associated with malnutrition, as individuals with lower BMI may have weaker immune function, metabolic capacity, and self-regulation compared to those with higher BMI within the normal range (35, 36). Therefore, this group of people may have a higher risk of death. In addition, in the baseline data of patients, we observed that those in the low TyG-BMI group had a higher average age and lower insulin levels. Previous studies have indicated that exogenous insulin can dilate and protect blood vessels, as well as alleviate myocardial ischemia-reperfusion injury (37, 38). Nevertheless, the association between endogenous high-level insulin and CVD requires further investigation.

### Advantages and limitations

Our research possesses several advantages. Firstly, we screened specific populations suffering from CVD, encompassing coronary heart disease, angina pectoris, myocardial infarction, and heart failure. CVD, being a common chronic illness and a leading cause of death posing significant harm, if its mortality risk can be predicted and intervened upon early, it will significantly alleviate the burden on the global public health system. Secondly, this study used the NHANES database, and the conclusions were representative to a certain extent. The influence of different covariates was fully adjusted in the statistical process. For missing data less than 15%, multiple imputation was used to supplement them, so that the interpolated data remained the same as the standard deviation of the mean to avoid bias caused by excessive deletion of missing data.

However, our research also has some limitations. Like other cross-sectional studies, this study was unable to establish a causal relationship between TyG-BMI index and all-cause mortality. In addition, although we have tried our best to adjust for potential confounding factors in the process of constructing statistical models, there are still many residual factors that have not been measured or included, which have a certain impact on the results. In addition, due to limitations in NHANES data, we were unable to obtain specific causes of death for some patients, thus preventing us from conducting in-depth analysis.

### Conclusion

In summary, this study found a significant association between TyG-BMI index and all-cause mortality in patients with CVD. Compared with TyG, TyG-BMI can be used as a predictive indicator of all-cause mortality in CVD patients, and has good specificity and sensitivity.

## Data Availability

The datasets presented in this study can be found in online repositories. The names of the repository/repositories and accession number(s) can be found below: https://www.cdc.gov/nchs/nhanes/.
